# Prevention of Recurrent Cases of Emergence Delirium in Electroconvulsive Therapy

**DOI:** 10.7759/cureus.106057

**Published:** 2026-03-29

**Authors:** Mohamad I Saeed, Christoffer K Kjærgård Hansen, Anders Jørgensen, Lone Baandrup

**Affiliations:** 1 Psychiatry, Bispebjerg-Gentofte, Mental Health Centre Copenhagen, Copenhagen, DNK; 2 Medicine, Institute of Clinical Medicine, University of Copenhagen, Copenhagen, DNK

**Keywords:** depression, electroconvulsive therapy, emergence delirium, lithium, propofol, thiopental

## Abstract

Electroconvulsive therapy (ECT) is an effective treatment for severe and treatment-resistant depression but may be complicated by postictal/emergence delirium. We report the case of a 56-year-old woman with psychotic, treatment-resistant depression who developed severe emergence delirium after each of her first four ECT sessions performed under thiopental anesthesia, with the need for repeated administration of benzodiazepines and propofol to terminate symptoms. Concurrently, her lithium medication dose was gradually reduced. After switching the anesthetic agent from thiopental to propofol at the fifth session, emergence delirium ceased, and subsequent treatments were well tolerated with adequate seizures in all but one session. This case suggests propofol anesthesia may help prevent emergence delirium after ECT, though its independent effect remains uncertain, and further studies are needed to investigate this.

## Introduction

Electroconvulsive therapy (ECT) is a safe, effective, and essential treatment for several serious psychiatric disorders, including particularly severe and treatment-resistant depression [1]. However, in up to 12% of cases, postictal/emergence delirium is observed [2]. This condition may be caused by the induced seizure activating a neural focus with abnormal activity and is characterized clinically by motor agitation, clouded consciousness, and disorientation to a severe degree that does not resolve within minutes, as is usually observed with postictal confusion [3]. Clinical consequences of emergence delirium following ECT include patient distress, risk of harm, need for additional sedation, and potential interruption of the ECT series [2]. One of the risk factors associated with this condition includes concurrent use of lithium; Patel et al., in a large register-based study in the USA, found 11.7-fold higher odds of emergence delirium compared to ECT without concurrent use of lithium [4]. Other reported risk factors for emergence delirium are catatonia [5], cerebrovascular disease [6], Parkinson’s disease [6,7], dementia [6,8], bilateral electrode placement [6], and longer ECT seizures as well as stimulus intensity [9]. Nevertheless, these studies are few and limited by small sample sizes, which complicates drawing conclusions regarding definite predictors of emergence delirium after ECT.

To reduce the risk of adverse events and enhance the quality of ECT, patients are administered anesthetic agents such as thiopental, propofol, etomidate, and methohexital; however, the preference for which agent to use is unclear [10]. A Cochrane meta-analysis suggests that methohexital or etomidate may be superior to thiopental or propofol in terms of eliciting an adequate seizure and that propofol may allow faster recovery time compared to thiopental, although the included studies were of limited quality and subject to bias [10]. In addition, the choice of anesthetic agent could vary based on the adverse effect profile, regional availability, clinician experience, and institutional protocols [10, 11]. There is limited data on the effects of switching the anesthetic agents during an ECT series [12], especially in regard to the prevention of emergence delirium. We present a case report in which a patient experienced emergence delirium after each session of ECT until thiopental was replaced with propofol.

## Case presentation

A 56-year-old woman with a history of periodic depression was admitted to the Mental Health Centre Copenhagen for ECT due to worsening, severe depression. Beforehand, treatment with medication and repetitive transcranial magnetic stimulation had been attempted. The patient was receiving pharmacological treatment with isocarboxazid 50 mg, lamotrigine 300 mg, nortriptyline 50 mg, lithium carbonate 900 mg, and quetiapine 325 mg daily.

Prior to admission, the patient had experienced worsening depressive symptoms over several months, including intrusive feelings of guilt, fleeting suicidal thoughts, and delusions that her spouse was unfaithful to her. Objectively, she appeared distressed with psychomotor retardation and depressed mood. The condition was assessed as psychotic depression. During a previous episode of severe depression, the patient had received 19 bilateral and five right unilateral ECT-sessions and experienced prolonged cognitive side effects. During the current hospitalization, right unilateral ECT was therefore prescribed with three treatments per week with energy at 55% for the first session, in accordance with the department's instructions. Lamotrigine was reduced to 50 mg daily, and the patient had a plasma lithium level of 0.75 mmol/L (ref. range: 0.5-0.8 mmol/L) on the day of the first session.

The body weight of the patient was 64.9 kg; thus, thiopental 250 mg and suxamethonium 40 mg were used for anesthesia during the first four ECT sessions. After each of these treatments, the patient woke up from anesthesia in an agitated state lasting 30 to 45 minutes with fluctuating awareness, screaming, and acting out with a need for physical restraint, thus in emergence delirium (Table 1). After the first session, an attempt was made to break the condition with diazepam 10 mg given twice, and after the second session, with diazepam 10 mg given four times as well as propofol 40 mg. After the third session, the attempt to break the delirium was with diazepam 10 mg given four times, as well as propofol given, respectively, at 40 mg and 20 mg. After the fourth session, midazolam was given as 4 mg and 1 mg; propofol was given, respectively, as 40 and 30 mg once, plus 20 mg given three times (Table 1). After the delirium, the patient was quite sedated by the administered medications with slightly impaired respiration and a need for brief staff assistance for airway maneuvers, although without supplemental oxygen. To prevent emergence delirium, the patient's usual dose of lithium carbonate was gradually reduced from 900 to 300 mg after the first session. Plasma lithium levels were 0.41 mmol/L at the fourth session and 0.28 mmol/L at the seventh treatment (reference range: 0.5-0.8 mmol/L).

**Table 1:** Overview of the patient's ECT ECT: Electroconvulsive therapy, Sux: Suxamethonium

ECT session no.	Anesthesia	Seizure duration	Emergence delirium	Accumulated post-ECT medication	Dosage of lithium carbonate	Plasma lithium	Reference range for plasma lithium
1	Thiopental 250 mg + suxamethonium (Sux) 40 mg	67 seconds	Yes	Diazepam 20 mg	900 mg daily	0.75 mmol/L	0.5-0.8 mmol/L
2	Thiopental 250 mg + Sux 40 mg	39 seconds	Yes	Diazepam 40 mg + propofol 40 mg	600 mg daily	-	-
3	Thiopental 250 mg + Sux 40 mg	38 seconds	Yes	Diazepam 40 mg + propofol 60 mg	600 mg daily	-	-
4	Thiopental 250 mg + Sux 40 mg	43 seconds	Yes	Midazolam 5 mg + propofol 130 mg	600 mg daily	0.41 mmol/L	0.5-0.8 mmol/L
5	Propofol 120 mg + Sux 40 mg	23 seconds	No	None	300 mg daily	-	-
6	Propofol 110 mg + Sux 40 mg	19 seconds	No	None	300 mg daily	-	-
7	Propofol 180 mg + Sux 40 mg	Inadequate	No	None	300 mg daily	0.28 mmol/L	0.5-0.8 mmol/L

**Figure 1:**
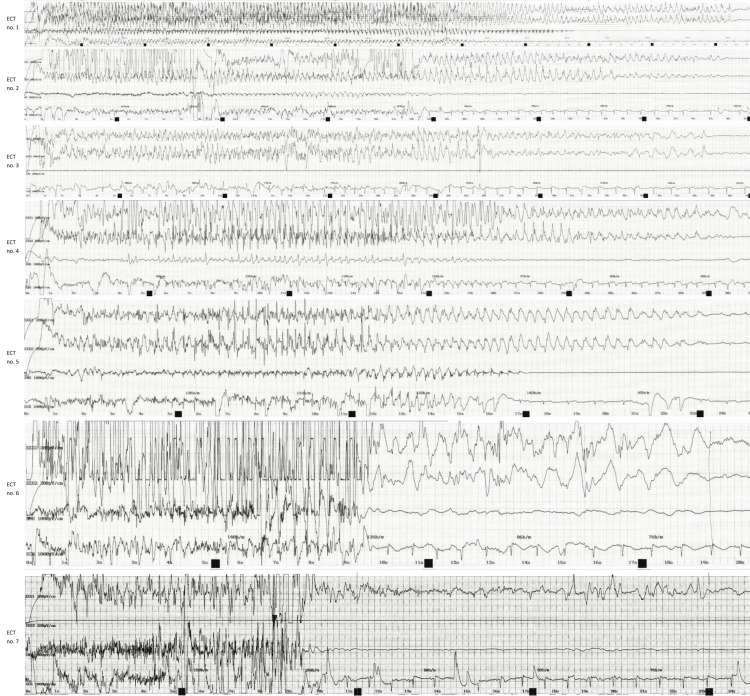
Ictal electroencephalography (EEG) strips from the patient's ECT sessions Each strip has four lines: EEG 1, EEG 2, electromyography (EMG), and electrocardiogram (ECG). Time is measured in seconds along the x-axis, and each strip shows the entire duration of the seizure from stimulus to termination. Note: the first six sessions of ECT yielded adequate seizures with rapid-onset, high-amplitude, slow-wave activity, progressing to a more regular ictal slow-wave pattern and ending with a high degree of postictal suppression, while the seventh session yielded an inadequate seizure. ECT: Electroconvulsive therapy, EEG: Electroencephalography

At the fifth ECT-session, anesthesia was initiated with propofol 120 mg instead of thiopental, and thereafter the patient woke up after each session without emergence delirium (Table 1). The propofol dose was adjusted depending on the degree of sedation. The first six ECT sessions were all characterized by electroencephalographically sufficient seizures in accordance with guideline standards, with a clear structure of rapid-onset, high-amplitude slow-wave activity, progressing to a more regular ictal slow-wave pattern and ending with a high degree of postictal suppression (Table 1 and Figure 1). The seventh session was assessed as an inadequate seizure, missing the aforementioned characteristics (Table 1 and Figure 1).

After the ECT series, the patient reported feeling joy, improved energy levels, and that her delusions had subsided. Objectively, she appeared to have improved in terms of psychomotor tempo and mood. However, she complained of cognitive side effects, including anterograde and retrograde amnesia as well as difficulty concentrating, and did not want further sessions of ECT.

## Discussion

This case raises the possibility that using propofol rather than thiopental may be associated with reduced emergence delirium in select patients, although concurrent lithium reduction and other factors prevent any causal inference. In lithium-treated patients, a reduction in dose to a plasma level of around 0.4-0.5 mmol/L is recommended, as the combination of ECT and lithium has been associated with a higher incidence of emergence delirium [4,13]. As the lithium carbonate dose in our case report was gradually reduced at the same time as the switch from thiopental to propofol, it cannot be determined with certainty whether propofol alone would have had the same prophylactic effect against emergence delirium. Additionally, other factors could explain the resolution of the emergence delirium from the fifth session of ECT, e.g., the progressive reduction of lithium to subtherapeutic levels, the cumulative effects of repeated benzodiazepine and propofol administration in earlier sessions, or possible changes in the patient’s underlying psychiatric state over time.

Propofol as prophylaxis against emergence delirium has only been described in a case report regarding a patient treated with clozapine and ECT for schizophrenia [14]. The authors found propofol to be effective in preventing postictal delirium both when used before ECT as anesthesia and immediately after seizure end [14]. The use of propofol for treating emergence delirium post-ECT has been reported to be effective [5,15] although it has likely contributed to the patient’s varying sedation and slightly impaired respiration with a brief need for staff assistance for airway maneuvers. Furthermore, the anticonvulsant properties of propofol may potentially counteract effective seizures during ECT [10]. Nevertheless, the fifth and sixth sessions of ECT yielded adequate seizures with the use of propofol, but careful titration and monitoring remain essential, and the inadequate seizure at session seven illustrates the potential challenge. All in all, this case suggests that propofol could be considered a relevant choice in select patients with recurrent/severe cases of emergence delirium when standard measures such as benzodiazepines are insufficient, and emphasizes the need for larger systematic studies to clarify its effect.

## Conclusions

This case suggests that replacing thiopental with propofol for anesthesia during ECT could be considered to help reduce the risk of emergence delirium. The patient developed severe delirium after each session with thiopental, which resolved completely after switching to propofol, allowing continuation of ECT and clinical improvement of psychotic depression. Given the concomitant reduction in lithium levels, this single case cannot establish causality. However, it may encourage further systematic evaluation of propofol in select patients with recurrent or severe emergence delirium after ECT.
